# 

**Published:** 1892-04

**Authors:** 


### THE MEDICAL ASSOCIATION.

The Forty-third Annual Session of the Georgia Medical Association meets in Columbus on the 20th instant. We hope there will be a large attendance of the profession from all over the State, and that much good will accrue from the assembling of the doctors together.

We may suggest one subject which we would be pleased to have the Association consider in its deliberations, and that is, the question of the establishment of a Board of Health, or Board of Medical Examiners, or some Board, whose function and powers shall be to determine who shall practice medicine within the State. This is an important matter, but it does not seem to be readily appreciated.

A good programme has been arranged, and we are sure the Columbus meeting will be pleasant, profitable and successful. We will be there.

PRELIMINARY PROGRAMME FORTY-THIRD ANNUAL SESSION OF THE MEDICAL ASSOCIATION OF GEORGIA, TO BE HELD IN COLUMBUS, GA., APRIL 20TH, 21ST AND 22D, 1892.—*Papers to be Read:* Partial list. The President's Annual Address, G. W. Mulligan, M. D., Washington; Cough—Some of its Causes and Treatment, Chas. D. Roy, A. B., M. D., Atlanta ; So-Called Typho-Malarial Fever, W. P. Williams, M. D., Waycross ; Preliminary Observations on the Behavior of Iodine in the Presence of Camphor, Menthol, Thymol, etc., R. J. Nunn, M. D., Savannah ; Some Observations upon Cataract Operations and After Treatment, A. W. Calhoun, M. D., Atlanta ; Remittent Fever, A. C. Blain, M. D., Brunswick ; Extirpation of Rectum for Carcinoma, J. McF. Gaston, M. D., Atlanta ; Some of the Fads and Fancies of the Medical Profession, J. C. LeHardy, M. D., Savannah ; Treatment of Pneumonia, with Report of Cases, H. Perdue, M. D., Barnesville ; How Shall We Manage the

Uterus after Abortion? K. P. Moore, M. D., Macon; Plaster of Paris in Surgery, W. F. Westmoreland, M. D., Atlanta; Report of Surgical Cases from my Note Book, J. B. Hinkle, M. D., Americus; What is Gynecology? R. R. Kime, M. D., Atlanta; A Case of Ovarian Cysts, J. M. Spence, M. D., Waresboro; Some Remarks on Tonsil Excisions, with Presentation and Description of a New Instrument, A. G. Hobbs, M. D., Atlanta; How to Best Conduct Labor to Prevent Injuries to the Os Uteri and Perineum, A. W. Griggs, M. D., West Point; Gunshot Wounds of Eye—Unusual Results, Geo. A. Wilcox, M. D., Augusta; The Treatment of Abortion and Some of the Complications, Walter A. Crow, M. D., Atlanta; Report of Case of Catalepsy and Treatment, Albert Sydney Johnson, M. D., Bowman; Typhlitis and Report of Case, S. M. Mathews, M. D., Quitman; The Relations and Dependencies Existing between the Specialist and General Practitioner of Medicine and Surgery, J. W. Griggs, M. D., West Point; The Relation between Skin Diseases and the General Health, M. B. Hutchins, M. D., Atlanta; Treatment of Hæmorrhoids by Carbolic Acid Injection, J. W. Hallum, M. D., Carrollton; Hemeralopia or Night Blindness, S. Latimer Phillips, M. D., Savannah; A Combination of Carbolic Acid and Camphor as an Antiseptic, Wm. Perrin Nicolson, M. D., Atlanta; Chorea, Hugh Hagan, M. D., Atlanta; Antiseptic Surgery, Ralph E. Smith, M. D., Atlanta; Radical Surgery the Best Surgery in the Treatment of Extensive Lacerated and Contused Wounds of the Extremities, E. H. Richardson, M. D., Atlanta; Typho-Malarial Fever, J. W. Duncan, M. D., Atlanta; The Action of Fibroid Tumors after the Menopause, Virgil O. Hardon, M. D., Atlanta; Intestinal Obstruction—Varieties, Diagnosis and Treatment, J. B. Hinkle, M. D., Americus; Report of Perineal Sections for Stricture, Stone in Bladder and Cystitis, Floyd W. McRae, M. D., Atlanta; Supra-pubic Lithotomy, with Report of Cases, W. S. Elkin, M. D., Atlanta; Typhlitis, William O'Daniel, M. D., Bullard.

G. W. MULLIGAN, M. D.,
*President.*

DAN H. HOWELL, M. D.,
*Secretary.*

